# Editorial Notices

**Published:** 1888-03

**Authors:** 


### EDITORIAL NOTICES.

An esteemed friend asks us whether the notices of appliances, remedies, etc., which he sees in this journal, are purchased "puffs" or the expression of an honest and unbiased opinion. There is a sting in the query, although we are fully convinced that the writer intended no intimation that we were purchasable. It is such a common occurrence for journals to promise and to give to all advertisers "reading notices," usually prepared by the advertisers themselves, that we do not wonder that our friend may fear that we have fallen into the evil practice.

Five years ago the publishers of this journal gave notice that no advertisement would be accepted which they have not cause for believing worthy the attention of honest men. They have tried to live up to that rule, and there has scarce been a month since in which they have not declined to admit advertisements of articles or firms that they could not conscientiously commend. In one case a page advertisement, for which a profitable contract had been made, was inserted three months before it was discovered that we had been imposed upon by false representations, yet it was at once removed and the amount forfeited, although by allowing it to stand for another month we might have received a considerable sum of money upon the contract. If among our advertisers there is a single firm that is not entirely reliable, or an article recommended that is not worthy of patronage, and the fact be demonstrated, that objectionable advertisement will be at once removed.

When the present editor assumed control of the INDEPENDENT PRACTITIONER, he announced that he would examine and make a practical test of any appliance or material that might be sent him, and if he believed it worthy he would give it notice in the journal. But this was upon condition that anything of material value, after it had been tested, would be returned to the sender at his expense, unless he desired it to remain on exhibition for his own benefit. This condition was insisted upon lest it might be thought that our opinion was unduly biased by a valuable present. The reasons for offering to examine and report upon new appliances, either personally or at the hands of one of our associates, were that many easily constructed little conveniences are devised by practitioners, and if they were described and commended when worthy it might assist many of our readers.

Again, it is well known that unprincipled men have patented devices which had originated with others, and in many instances dentists have been called upon to pay royalties upon some appliance which they had themselves originally conceived. By making a permanent record of their inventions this pirating would be effectually prevented. There is no doubt that had this course been pursued in the past, dentists would not now be subjected to the exactions of some greedy patentees. In this number four or five little conveniences are mentioned, either of which might be made by any dentist, and their pecuniary value is but a very few cents. Yet they are more gladly given space than they would be if they represented dollars, for they are mentioned in the sole interest of our readers.

No article or remedy has been or will be commended which some one of the publishers of this journal has not personally tested and approved. No manufacturing or mercantile firm has been or will be mentioned of whose entire reliability we are not fully assured. No person has yet had the hardihood to insult us by offering any valuable consideration for a favorable notice. Should any do so, they will be most emphatically convinced that our opinions are not in the market, and that if any scoundrel attempts to purchase the commendation of this journal it knows how properly to resent the affront.

We cannot expect every one to agree with us as to the value of an appliance or book, for what would be useful to one, another might pronounce worthless. Our opinions may not be of any particular importance, but they shall at least possess the merit of being honest convictions.

